# Character Strengths, Strengths Use, Future Self-Continuity and Subjective Well-Being Among Chinese University Students

**DOI:** 10.3389/fpsyg.2018.01040

**Published:** 2018-06-29

**Authors:** Yonghong Zhang, Mengyan Chen

**Affiliations:** School of Culture and Social Development Studies, Southwest University, Chongqing, China

**Keywords:** character strengths, strengths use, future self-continuity, subjective well-being, positive education

## Abstract

The study was designed to explore the relationships among character strengths, strengths use, future self-continuity and subjective well-being. A total of 225 undergraduates completed paper-and-pencil questionnaires assessing character strengths, strengths use, future self-continuity, and subjective well-being. Results suggested several character strengths were correlated with subjective well-being and the strongest correlations were found for hope, curiosity, zest, perseverance and love. All character strengths were significantly correlated with strengths use. Strengths use and future self-continuity were robustly correlated with subjective well-being. The mediation analysis showed that strengths use mediates the relationship between character strengths and subjective well-being, and specifically, the indirect effects of strengths use varies from different character strengths. The moderated mediator suggested that future self-continuity moderated the mediation of strengths use because future self-continuity moderates the effect of strengths use on subjective well-being. Furthermore, the indirect effect of strengths use was stronger with high level of future self-continuity than those with low level of future self-continuity. The present findings make a contribution to understand the underlying mechanisms involving in character strengths are associated with higher level of well-being. Additionally, the findings expand knowledge about future self-continuity and its relation to strengths use and subjective well-being among undergraduates, having significant implications in the educational context.

## Introduction

Over the last decade, worldwide there is a growing interest in positive psychology and its emerging applied field of positive education from the youngest years through to university students. Given the shockingly high prevalence of psychological disorder or depression among adolescents and young adults, psychologists, educators or policy makers became aware that, apart from equipping students with knowledge and skills for accomplishment, success, literacy and discipline, education should also teach students well-being by adopting findings of positive psychology (Seligman et al., 2009). In other words, the end-goal of schooling is to help students not only function well but also feel good. One central concern of positive psychology is character strengths. According to Peterson and Seligman (2004), character strengths can be recognized as an entire cluster of positive traits vital for the good life, manifesting through a range of thoughts, feelings and behaviors. Moreover, these strengths are morally valued and universal, while there are individual differences that exist in degrees and vary across the lifespan (Park and Peterson, 2006a). In order to approach the multidimensionality of character strengths and assess these strengths as individual differences, based on the existing scientific literature, philosophical tradition and historical surveys, Peterson and Seligman (2004) introduced the classification of Virtue in action (VIA) which identifies 6 core virtues and the 24 character strengths. Also, Peterson and Seligman suggested that there are several criteria for the inclusion of character strengths in the classification, and one of these criteria is to contribute to the fulfilling life and happiness (Park et al., 2004).

A large body of literature has developed showing that possessing character strengths are correlated with various indicators of subjective well-being (SWB) (Park et al., 2004; Park and Peterson, 2006b; Shimai et al., 2006; Gillham et al., 2011; Shoshani and Slone, 2013; Weber et al., 2013; Martínez-Martí and Ruch, 2014). In a preliminary survey of the relationship between character strengths and SWB, participants were asked to complete the VIS Inventory of strengths (VIA-IS) and Satisfaction with Life scale (SWLS), and the results showed that the five strengths, namely, hope, zest, gratitude, love and curiosity, had the strongest correlations with life satisfaction, and modesty, creativity, judgment, appreciation of beauty, love of learning were the least correlated with life satisfaction, and also provided the empirical evidence that “the higher a given strength, the more life satisfaction” (Park et al., 2004). Of note, the finding that hope, zest, gratitude, love and curiosity are the best predictors of life satisfaction has been replicated in most of the subsequent research across different cultures (Shimai et al., 2006; Peterson et al., 2007; Brdar and Kashdan, 2010; Ruch et al., 2010, 2014; Zhou and Liu, 2011; Buschor et al., 2013). Such findings have demonstrated the convergence and similarity in terms of the relationship between character strengths and satisfaction of life to a large extent.

Literature also illustrated positive associations between character strengths and positive affect (PA) (Van Eeden et al., 2008; Littman-Ovadia and Lavy, 2012; Shoshani and Slone, 2013; Weber et al., 2013; Martínez-Martí and Ruch, 2014). Martínez-Martí and Ruch (2014) found that the strengths of hope, zest, humor, gratitude and love yielded the highest correlation with PA, whereas the lowest correlations were modesty, religiousness, appreciation of beauty and excellence, prudence, and open-minded. Also, Littman-Ovadia and Lavy (2012) suggested that hope, curiosity, zest, love of learning, and perspective were the five strengths most strongly and positively related to PA, whereas modesty, forgiveness, and spirituality were the ones correlated lowest (non-significant, *all*). In relation to negative affect, Martínez-Martí and Ruch (2014) suggested that hope, humor, zest, honesty and open-mindedness were the ones with mostly highest negatively correlations, whereas appreciation of beauty and prudence were the ones with the lowest negative correlations. Littman-Ovadia and Lavy (2012) demonstrated that hope, curiosity, zest, love, and self-regulation were the ones with mostly highest negatively correlations, whereas excellence, modesty, creativity, bravery and prudence were the ones with the lowest negative correlations.

In addition, there were other existing studies investigating the relationship between character strengths and other indicators of subject well-being (e.g., happiness, job satisfaction, and quality of life) and other domains of well-being such as psychological well-being (PWB; Park and Peterson, 2006b; Buschor et al., 2013; Hausler et al., 2017; Park and Lee, 2017). In particular, Hausler et al. (2017) examined the different relations between individual character strengths and two types of well-being, namely, SWB and PWB, and found that the strengths of hope, zest, gratitude, curiosity and love (*called* “the happiness strengths”) were related mostly to PWB and SWB and the 24 character strengths in general showed the stronger correlations with PWB than SWB. Accordingly, it can be concluded that the possession of character strengths can be significantly and robustly associated with positive functioning. However, there is a question occurring, that is, how these strengths operate to promote positive functioning and well-being?

According to Seligman and Csikszentmihalyi (2000), exerting and habituating one’s character strengths allows people to experience a sense of fulfillment and results in a satisfying life. Similarly, Peterson and Seligman (2004) suggested character strengths use is related to a feeling of self with vigor and authenticity, and leads to positive functioning and well-being (Proctor et al., 2011a). That is, when a person uses his strengths, he feels as if he has more energy, being more vigorous and alive, and feels as if he can reveal his true self, more authentic and engaging, resulting in positive functioning and performance (Linley, 2008; Dubreuil et al., 2014). Cross-sectional and longitudinal literature has demonstrated the positive link between strengths use and well-being (Linley et al., 2010; Proctor et al., 2011a; Wood et al., 2011; Harzer and Ruch, 2013; Botha and Mostert, 2014; Douglass and Duffy, 2015; Huber et al., 2017; Littman-Ovadia et al., 2017). In a longitudinal study, for example, strengths use was found to be significantly and positively associated with self-esteem, vitality, PA, and predicted increased well-being over time (Wood et al., 2011). Another study with undergraduate students also suggested that strengths use and satisfaction with life yielded a significant and positive relation (Douglass and Duffy, 2015). Moreover, in the work setting, researchers examined the associations among three types of strengths use and work outcomes. Results showed that signature-strengths use, happiness-strengths use, and lowest-strengths use had beneficial effects on different aspects of work-related outcomes. That is, using signature strengths (SS) was mostly correlated to behavioral aspects of functioning [e.g., performance, organizational citizen behavior (OCB), counterproductive work behavior (CWB)] at work; using happiness strengths was mostly correlated to emotional-psychological aspects including job satisfaction and work engagement and work meaningfulness; and using lowest strengths held unique contribution to OCB, and although using different kinds of would be beneficial, happiness-strengths use can be particularly beneficial for most people (Littman-Ovadia et al., 2017).

Moreover, many intervention studies also suggested that using one’s character strengths has consistently and positively correlated with various domains of well-being (Seligman et al., 2005; Mitchell et al., 2009; Sin and Lyubomirsky, 2009; Quinlan et al., 2012; Bolier et al., 2013; Proyer et al., 2015; Harzer and Ruch, 2016; Meyers and van Woerkom, 2017). For example, in the first strength-based intervention study, participants was asked to use one of their top five strengths in a new way each day over 1 week, and the obtained results showed that participants did experience significantly greater benefits in well-being over a period of 6 months (Seligman et al., 2005). This design “using the top 5 strengths” or “using SS” has also become the main strategy in the subsequent intervention research (Mitchell et al., 2009; Proctor et al., 2011b; Bridges et al., 2012; Mongrain and Anselmo-Matthews, 2012; Gander et al., 2013; Duan et al., 2014; Proyer et al., 2014). Of note, there also have a small number of interventions studies focusing on lesser strengths (LS) or weaknesses (Rust et al., 2009; Walker, 2013; Proyer et al., 2015). For instance, in the work of Proyer et al. (2015), 375 adults were randomly assigned to a LS intervention, a SS intervention, and a placebo control condition. The results showed that participants in both intervention conditions reported increases in happiness lasting 3 months and decreases in depressive symptoms in the short term and the intervention targeting the LS led to significant improvement in satisfaction with personal health and quality of living conditions in general.

Therefore, it may be concluded that the possession of strengths is a prerequisite of a fulfilling life and positive functioning; whereas the unblocked deployment is the direct way to achieve a good life. In fact, there is a clear difference between possessing strengths and using strengths. For instance, if a person was highly creative but never makes use of this strength, he is unlikely to get much benefit from this strength. In contrast, a person has high level of creativity and make the most of such a strength in different ways such as completing required tasks creatively in the workplace, he will get the most benefit such as experiencing a strong sense of accomplishment (Wood et al., 2011). Consistent with this argument, in a previous cross-sectional study, the authors found that although knowledge about people’s strengths and strengths use were both highly correlated with indicators of well-being, they had distinctly predictive effects on well-being, Specifically, knowing one’s strengths would not cause any significant increases in well-being, while using strengths promoted stronger vitality and well-being (Govindji and Linley, 2007).

Taken together, the positive link between the possession of character strengths and well-being may be built with making use of personal strengths. Indeed, for individuals when they authentically apply their strengths in a wide variety of daily situations, they can experience a sense of fulfillment and achievement, resulting in the achievement of happiness (Peterson and Seligman, 2004). However, to the best of knowledge, almost no research has directly examined the role of strengths use in relation between the possession of character strengths and well-being. Therefore, the leading purpose of the current research is to examine the mediating role of strengths use in the link between character strengths and SWB, and further investigate the indirect effect of every strength on SWB through the “bridge” of strengths use.

According to the temporal model of SWB proposed by Durayappah (2011), the definition of global SWB not only consider the immediate thoughts and states as well as the present self, but also concerns the past, future thoughts and states as well as past and present selves. As such, the temporal state is a fundamental component in the model of SWB because when people evaluate their global life satisfaction, they consider “not only current proceedings, but also the moments that have occurred, as well as those yet to be.” Although we live in the present, we often recall the past life stories and also anticipate the future events; such kinds of imagination, connecting the past, present and future selves into a continuous narrative, stems from the perceived personal “sameness” over time, a sense of connectedness and similarity to the temporal selves through time (James, 1985; Blouin-Hudon and Pychyl, 2016). The sense of self-continuity is vital throughout the life ranging from building up social networking to interpreting the surrounding world and to making emotional responses for that world as well as planning for the future (Sadeh and Karniol, 2012).

On the basis of motivated identity construction theory, the self-continuity perspective plays a key role in how people develop and maintain their senses of identities that strongly influence personal and societal functioning (Bird and Reese, 2008; Vignoles et al., 2011; Becker et al., 2017). According to Murillo (2012), every moral behavior might be based on a conception of the self over the course of time, which is compatible with the direction of self-realization, and the conceptualization of personal identity involving the continuity of the person over the course of time has close relationships with the general goal of life, namely, happiness. Similarly, Konut suggested that a feeling of personal continuity is related to greater creativity, vitality and self-esteem. Furthermore, prior psychologists suggested that the perception of the temporally extended self is a central part of self-knowledge (James, 1890/1981; Stern, 1985), which can be seen as an important predictor of global well-being and psychological health (Bénabou and Tirole, 2003; Kurtz, 2011; Ahadi et al., 2015). Empirical studies on self-continuity showed that individuals’ feeling of connected and continuous self has robust impacts on a range of consequential outcome including time management, decision making, saving behavior, well-being and coping (Singer and Bluck, 2001; Ersner-Hershfield et al., 2009a, 2012; Sadeh and Karniol, 2012; Blouin-Hudon and Pychyl, 2015). For example, Chandler et al. (2003) demonstrated that a sense of personal continuity over time enables adolescents to show appropriate care and concern for themselves and promotes well-being in the long term.

In many discussions of self-continuity, the sense that the present self connects to the future, namely, future self-continuity is of considerable concern. According to Parfit (1971), if a person recognizes his future self as a stranger, he might have no more reasons to work on his resources for his future self than for this stranger; conversely, if he perceives the future self as similar to the present self, he is more likely to make prudent decision. On a more general level, individuals’ feelings of connectedness to the future self affect one’s attitude and behavior. For example, for people who have greater continuity with their future self, they are more willing to save for the future and place a high value on future gains (Ersner-Hershfield et al., 2009a,b). Similarly, when the future self shares similarity to the present self with a vivid and realistic terms and in the positive affective state, people might be more likely to make sacrifices today that may benefit them at some point in the years to come (Ersner-Hershfield, 2011). Along with these findings, Adelman et al. (2017) suggested that future self-continuity also plays a signature role in the educational setting and has beneficial impacts on academic performance, by considering more possible and long-term consequences instead of merely focusing on the current and short-term consequences. Additionally, the perceptions of future self-continuity may have a positive correlation with positive affective states and specifically, people with high level of future self-continuity might feel pleasant when they imagine how the current behavior may cause positive future consequences, and in turn, those experiencing greater positive affection might be more willing to include the patterning of future self’s goals (Blouin-Hudon and Pychyl, 2015).

Based on these considerations, we conclude that future self-continuity plays an important role in initiating one’s rational behavior and is closely related to his or her affective states, and for those high in future self-continuity, they are more likely to exhibit the beneficial action (e.g., applying strengths in different ways) and draw attention on the positive long-term consequences of their beneficial action, and in turn experience the positive affective state. To the best of our knowledge, almost no prior studies have examined the role of future self-continuity in the relationships between strengths of character, strengths use and SWB. Thus, the second objective of the present study is to explore whether people with different levels of future self-continuity display different levels of strengths use and experience different levels of well-being from using their strengths, or alternatively, does the way in which strengths use mediates the association between strengths of character and SWB depend on one’s different levels of future self-continuity?

## The Present Study

The current study attempts to explore the relations among character strengths, strengths use, future self-continuity and SWB with a sample of Chinese undergraduate students. Specifically, this study has two objectives: (1) to examine the mediating role of strengths use in the relationship between the 24 character strengths and SWB and assess the indirect effect of each character strength on SWB through the “bridge” of strengths use; (2) to explore the moderating role of future self-continuity in the link of character strengths and SWB and the link of strengths use and SWB.

### Participants

A sample of 238 undergraduate university students from Southwest University participated in this survey. Thirteen students were excluded from the analysis because of incomplete data. Among the remaining 225 students, 52 (23.1%) were males and 173 (76.9%) were females. The average age of students was 19.23 years (*SD* = 0.816; range from 17 to 22). The sample was comprised of freshmen (47%), sophomores (32.6%), juniors (15.9%), and seniors (4.5%). With regard to students’ hometown, 78 (34.8%) came from rural areas, 83 (37.1%) came from small towns, and 63 (28.1%) came from large and medium-sized cities. One participant did not provide information about his hometown.

### Procedure

The present study received the Ethics approval from the school of Culture and Social Development in Southwest University of China. Our study also obtained the consent of the undergraduate students. Before the application of the questionnaires, participants were informed about the purposes of the present study. Alternative options were provided if participants did not wish to participate in the study. The study group also confirmed that all data would be kept confidential, only accessible to the study group and be only used for study purposes as well as there was no right or wrong answer. The data of the study were collected in the course of regular class hours.

### Instruments

#### Character Strengths

The *Chinese virtues questionnaire (CVQ)*, which was developed by Duan et al. (2012, 2013), was used in the present study to measure the 24 character strengths. CVQ is a simplified Chinese self-report questionnaire that measures the 24 widely valued character strengths and 3 virtues (i.e., interpersonal, vitality and cautiousness) with 96 items. An example of items is “I see beauty that other people pass by without noticing” (beauty). The respondents were asked to rate the extent to which each item described them on a five-point Likert scale ranging from 1 (very much unlike me) to 5 (very much like me). The means scores of the 24 strengths were obtained by summing the corresponding items of per strengths and then dividing them by the numbers of item. A high score represents a high degree of the character strength within an individual. CVQ showed good psychometric characteristic and solid cultural foundations in the existing research. The internal coefficient alpha were 0.90 (the interpersonal subscale), 0.91 (the vitality subscale), 0.88 (the cautiousness subscale), as reported by Duan et al. (2013), and the test-retest reliability for three subscales over 10 weeks ranged from 0.70 to 0.76 and the convergent validity ranged from 0.27 to 0.52, and also this scale showed clear discriminant validity from related constructs such as hope and gratitude. In another study, the reliability for this total questionnaire was 0.945 and the test-retest reliability over 6 weeks for three scales ranged from 0.738 to 0.826 and convergent validity ranged from 0.379 to 0.587 (Zhang et al., 2014). In the present study, the internal consistency coefficient of the total questionnaire was 0.96.

#### Strength Use

The *Strength Use Scale (SUS)*, which was developed by Govindji and Linley (2007), was used to assess individual strength use with 14 items. An example of items is “I always play to my strengths.” Participants were asked to each item on a seven-point Likert scale ranging from 1 (strongly disagree) to 7 (strongly agree). The higher the mean score of the whole scale reflected the higher degree of strength use. In the original study, the coefficient alpha of the SUS, as reported by Govindji and Linley (2007), was 0.95 and this construct has been demonstrated to associate significantly with other criterion measures including self-esteem, self-efficacy, and subjective vitality and with measures of related constructs including SWB, and PWB. Wood et al. (2011) suggested that this scale has good internal consistency (α_(T1/T2/T3)_ = 0.97/0.97/0.94) and test–retest reliability (*r* = 0.85) as well as good criterion validity with well-being. In the present study, the internal consistency coefficient was 0.93.

#### Future Self-Continuity

The *Future Self-continuity scale*, which was developed by Ersner-Hershfield et al. (2009b), was used to assess similarity between current and future selves. This scale was based on the inclusion of the other in the self scale (Aron et al., 1992). As such, the index of future self-continuity is measured by 2 questions on a 7-point scale marked at each point by two circles that ranged from depicting no overlap to depicting almost complete overlap. The first question asks participants to select the circle pair that best described how similar they felt to a future self 10 years from now on the scale ranging from 1 (not similar at all) to 7 (completely similar). The higher scores reflected the more continuity with one’s future self. The test–retest reliability over a period of 2 weeks, as reported by Ersner-Hershfield et al. (2009b), was high (0.79 for similarity and 0.80 for connectness) and the similarity rating on this scale was significantly correlated with other corresponding measures rating including match in adjective descriptions of present and future selves and valuation of future reward. Bartels and Urminsky (2011) suggested that the future self-continuity scale has clear discriminant validity from measures of related concepts such as uncertainty about the future and about one’s future preferences and general perceived change in life circumstances.

#### Subjective Well-Being

The *Positive and Negative Affect schedule (PANAS)*, which was developed by Watson et al. (1988), was used to assess positive and negative affectivity. The scale is made up of two subscales each consisting of ten items: 10 positive affects (i.e., interested, excited, strong, enthusiastic …) and 10 negative affects (i.e., distressed, upset, guilty, scared …). Participants were asked to rate the extent to which they had felt each feeling during the past week on a five-point Likert scale ranging from 1 (very slightly or not at all to extremely) to 5 (extremely). In the original study, the coefficient alpha of the PA and NA scales were 0.86 and 0.87, and the test-retest reliabilities after 1 week interval were 0.79 for PA and 0.81 for NA, and convergent validity ranging from 0.89 to 0.95 (Watson et al., 1988). The reliabilities for this scale, as also reported by Crawford and Henry (2004), was 0.89 for PA and 0.85 for NA, and the CFA results clearly supported the construct validity of the PANAS scales. In the present study, the internal consistency coefficients were 0.83 for PA and 0.87 for NA.

*Satisfaction With Life Scale (SWLS)*, which was developed by Diener et al. (1985), was used to measure global life satisfaction with 5 items. An example of items is “I am satisfied with my life.” Participants was asked to each item on a seven-point Likert scale ranging from 1 (strongly disagree) to 7 (strongly agree). The higher the total score of the whole scale reflected the higher level of global life satisfaction. In the original research, the coefficient alpha was 0.87and the test-retest correlation coefficient over a period of 2 months was 0.82 (Diener et al., 1985). Shevlin et al. (1998) reported that the common metric standardized factor loadings ranged from 0.92 to 0.98, and the reliability for this scale was 0.921. In the present study, the internal consistency coefficient was 0.76.

### Data Analysis

In order to explore the relationship among character strengths, strengths use, future self-continuity and SWB, we use SPSS 20.0 to conduct data analysis, which included four steps. As a first step, we computed descriptive statistics of 24 character strengths, and whether students’ age and gender were correlated with any variables analyzed for the present study questions with Pearson correlation. Because we observed that some character strengths would be influenced by participants’ gender, we decided to control for the effects of this demographic variables in the subsequent analyses. As a second step, we computed partial correlations between character strengths, strengths use, future self-continuity and SWB with gender as the control variable. As a third step, we tested whether strengths use mediated the relationship between the whole character strengths and SWB, and further examined the direct and indirect effects between 24 character strengths and SWB respectively with the help of an SPSS macro developed by Hayes (2013). As a final step, we explored the moderating role of future self-continuity in the link of character strengths and SWB and the link of strengths use and SWB, and further examined indirect effects of at different levels of the moderator.

## Results

### Preliminary Analyses

The results of the preliminary analyses are shown in **Table 1**. Means of 24 character strengths range from 2.90 (regulation) to 3.91 (fairness). The top five strengths were fairness, love, authenticity, gratitude as well as leadership, and the last five strengths were zest, creativity, perspective, learning as well as regulation, which are comparable with earlier findings. In addition, there were no significant correlations with age. Furthermore, compared with boys, girls were more likely to report higher scores on teamwork, fairness, Love, and gratitude. Because some of variables analyzed for the present study questions would be influenced by participants’ gender, we decided to control for the effects of this demographic variables in the subsequent analyses.

**Table 1:** Self-reported variables: means, standard deviations, and correlations with students’ age and gender.

Variables	*M*	*SD*	Correlations with	
			Age	Gender
Self-reported CVQ				
Kindness	3.70	0.57	0.055	0.092
Teamwork	3.73	0.57	0.010	0.137*
Fairness	3.91	0.51	–0.010	0.132*
Love	3.90	0.62	0.027	0.249**
Authenticity	3.84	0.52	0.014	0.095
Leadership	3.78	0.56	0.027	0.042
Forgiveness	3.61	0.62	0.025	0.062
Gratitude	3.79	0.58	–0.037	0.241**
Humor	3.23	0.75	0.023	–0.067
Curiosity	3.20	0.63	0.059	0.060
Zest	3.19	0.65	0.101	0.042
Creativity	3.19	0.67	0.001	–0.073
Perspective	3.16	0.58	0.043	–0.064
Hope	3.40	0.64	–0.020	0.064
Social	3.27	0.61	0.030	–0.124
Beauty	3.52	0.67	–0.031	0.129
Bravery	3.33	0.61	0.042	–0.027
Belief	3.24	0.70	0.031	–0.018
Judgment	3.44	0.58	–0.043	–0.148*
Prudence	3.50	0.62	0.017	–0.066
Regulation	2.90	0.60	0.016	–0.103
Perseverance	3.37	0.59	0.023	–0.049
Learning	3.10	0.74	0.081	–0.020
Modesty	3.24	0.57	0.053	0.025
SU	4.24	0.91	0.020	0.125
FSC	4.14	1.45	0.013	–0.013
SWB	4.06	1.60	–0.061	0.075

*N = 225. Age: 17–22 years. Gender: 1 = male, 2 = female. CVQ, Chinese virtues questionnaire; SU, Strengths use; FSC, Future self-continuity; SWB: Subjective well-being. ^∗^*p* < 0.05, ^∗∗^*p* < 0.01 (Pearson correlation, two-tailed).*

### Correlations

Partial correlations among study variables are shown in **Table 2**. All character strengths were significantly correlated with strengths use with the top coefficients being found for social, perspective, zest, creativity and humor, and 22 of the 24 character strengths were significantly related to SWB with the top coefficients being found for hope, curiosity, zest, perseverance and love, which was in line with previous findings (Park et al., 2004; Wagner and Ruch, 2015; Hausler et al., 2017). Forgiveness was not significantly related to students’ SWB, and prudence was negatively related to SWB. In addition, authenticity and perseverance were correlated with future self-continuity. Strengths use had no correlation with future self-continuity. Strengths use and future self-continuity were found to be correlated with SWB significantly. The significant correlations were exclusively positive.

**Table 2:** Partial correlations between 24 character strengths and strengths use, future self-continuity and subjective well-being.

Variables	SU	FSC	SWB
Kindness	0.346^∗∗^	0.060	0.247^∗∗^
Teamwork	0.385^∗∗^	0.131	0.270^∗∗^
Fairness	0.363^∗∗^	0.050	0.185^∗∗^
Love	0.356^∗∗^	0.083	0.321^∗∗^
Authenticity	0.434^∗∗^	0.156^∗^	0.209^∗∗^
Leadership	0.471^∗∗^	0.040	0.175^∗∗^
Forgiveness	0.201^∗∗^	0.082	0.089
Gratitude	0.316^∗∗^	0.037	0.151^∗^
Humor	0.503^∗∗^	–0.023	0.244^∗∗^
Curiosity	0.489^∗∗^	0.012	0.392^∗∗^
Zest	0.544^∗∗^	0.044	0.385^∗∗^
Creativity	0.528^∗∗^	–0.078	0.258^∗∗^
Perspective	0.568^∗∗^	0.056	0.189^∗∗^
Hope	0.406^∗∗^	0.026	0.466^∗∗^
Social	0.577^∗∗^	–0.043	0.286^∗∗^
Beauty	0.434^∗∗^	0.046	0.184^∗∗^
Bravery	0.441^∗∗^	–0.005	0.167^∗^
Belief	0.340^∗∗^	0.090	0.234^∗∗^
Judgment	0.466^∗∗^	0.024	0.146^∗^
Prudence	0.179^∗∗^	0.112	–0.010
Regulation	0.247^∗∗^	0.091	0.208^∗∗^
Perseverance	0.417^∗∗^	0.145^∗^	0.344^∗∗^
Learning	0.325^∗∗^	0.065	0.240^∗∗^
Modesty	0.243^∗∗^	0.158^∗^	0.201^∗∗^
			
SU	1.00	0.08	0.49^∗∗^
FSC	0.08	1.00	0.30^∗∗^
SWB	0.49^∗∗^	0.30^∗∗^	1.00

*N = 225. Correlations are controlled for influences of students’ gender. SU, Strengths use; FSC, Future self-continuity; SWB, Subjective well-being. ^∗^*p* < 0.05, ^∗∗^*p* < 0.01.*

### Mediation

We conducted a mediation analysis based on 5000 bootstrapped sample using bias corrected and accelerated 95% confidence intervals (CIs). Before the analysis, we z-transformed all the variables to ensure that variable effect sizes would be compared. As **Table 3** shown, character strengths had a significant, direct path to strengths use (*β* = 0.07, *SE* = 0.01, *p* < 0.01) in the mediator variable model. Next, when strengths use was included into the dependent variable model, the direct effect of character strengths on SWB was not significant (*SE* = 0.0136, 95% CI: {-0.0112, 0.0425}), and indirect effect was significant (*SE* = 0.0103, 95% CI: {0.0297, 0.0706}). Thus, strengths use mediates the relation between character strengths and SWB.

**Table 3:** Result of the mediation analysis of strengths use between the whole character strengths and subjective well-being.

Predictor	*B*	*SE*	*t*	*p*
**Mediator variable model**			
CCS	0.07	0.01	12.4677	0.0000
**Dependent variable model**			
CCS	0.02	0.01	1.15	0.2511
CM	0.73	0.13	5.46	0.0000

*CCS, character strengths; CM, strengths use.*

In addition, we examined the direct and indirect effects between 24 character strengths factors and SWB respectively. As **Table 4** shown, for most of the 24 character strengths (except forgiveness, judgment and prudence), the total effects were significant and positive, and hope was the strongest predictor of SWB with the regression weight 1.14^∗∗^.

**Table 4:** Results of mediation analyses for character strengths as predictors of subjective well-being with strengths use.

			Total effect	Direct effect	Mediation by strengths use	Total R^2^
		
	a	b	c	c’	indirect effect a × b	
Kindness	0.56^∗∗^	0.79^∗∗^	0.62^∗∗^	0.18	0.44^∗∗^	0.23^∗∗^
Teamwork	0.61^∗∗^	0.78^∗∗^	0.70^∗∗^	0.23	0.48^∗∗^	0.23^∗∗^
Fairness	0.65^∗∗^	0.84^∗∗^	0.49^∗^	–0.06	0.55^∗∗^	0.23^∗∗^
Love	0.55^∗∗^	0.74^∗∗^	0.78^∗∗^	0.38^∗^	0.40^∗∗^	0.24^∗∗^
Authenticity	0.75^∗∗^	0.84^∗∗^	0.59^∗∗^	–0.04	0.63^∗∗^	0.23^∗∗^
Leadership	0.77^∗∗^	0.90^∗∗^	0.46^∗^	–0.22	0.69^∗∗^	0.23^∗∗^
Forgiveness	0.31^∗∗^	0.84^∗∗^	0.18	–0.08	0.26^∗∗^	0.22^∗∗^
Gratitude	0.53^∗∗^	0.85^∗∗^	0.38^∗^	–0.06	0.44^∗∗^	0.23^∗∗^
Humor	0.59^∗∗^	0.84^∗∗^	0.48^∗∗^	–0.02	0.49^∗∗^	0.23^∗∗^
Curiosity	0.70^∗∗^	0.66^∗∗^	0.98^∗∗^	0.51^∗∗^	0.46^∗∗^	0.26^∗∗^
Zest	0.75^∗∗^	0.66^∗∗^	0.96^∗∗^	0.47^∗∗^	0.49^∗∗^	0.25^∗∗^
Creativity	0.67^∗∗^	0.86^∗∗^	0.50^∗∗^	–0.09	0.58^∗∗^	0.23^∗∗^
Perspective	0.87^∗∗^	0.95^∗∗^	0.49^∗∗^	–0.34	0.83^∗∗^	0.24^∗∗^
Hope	0.58^∗∗^	0.61^∗∗^	1.14^∗∗^	0.78^∗∗^	0.36^∗∗^	0.31^∗∗^
Social	0.83^∗∗^	0.84^∗∗^	0.67^∗∗^	–0.03	0.70^∗∗^	0.23^∗∗^
Beauty	0.60^∗∗^	0.85^∗∗^	0.45^∗∗^	–0.05	0.51^∗∗^	0.23^∗∗^
Bravery	0.65^∗∗^	0.88^∗∗^	0.41^∗^	–0.17	0.58^∗∗^	0.23^∗∗^
Belief	0.44^∗∗^	0.79^∗∗^	0.50^∗∗^	0.15	0.35^∗∗^	0.23^∗∗^
Judgment	0.67^∗∗^	0.91^∗∗^	0.32	–0.29	0.61^∗∗^	0.24^∗∗^
Prudence	0.25^∗^	0.86^∗∗^	–0.05	–0.26	0.21^∗^	0.24^∗∗^
Regulation	0.35^∗∗^	0.79^∗∗^	0.52^∗∗^	0.24	0.28^∗∗^	0.23^∗∗^
Perseverance	0.63^∗∗^	0.72^∗∗^	0.88^∗∗^	0.42^∗^	0.45^∗∗^	0.25^∗∗^
Learning	0.38^∗∗^	0.78^∗∗^	0.52^∗∗^	0.23	0.29^∗∗^	0.24^∗∗^
Modesty	0.39^∗∗^	0.80^∗∗^	0.52^∗∗^	0.21	0.31^∗∗^	0.23^∗∗^

*N = 225. a – Direct effect of IV (character strengths) on the mediator (strengths use); b – Direct effect of the mediator (strengths use) on DV (subjective well-being); c – Total effect of IV (character strengths) on DV (subjective well-being); c’ – Direct effect of IV (character strengths) on DV (subjective well-being); a × b – Indirect effect of IV (character strengths) on DV (subjective well-being) through the mediator (strengths use). *z* = 5000 bootstrap resamples. ^∗^*p* < 0.05, ^∗∗^*p* < 0.01.*

For all of the 24 character strengths factors, there were significant indirect effects (a × b), which means that the relationships between the 24 character strengths and SWB were mediated by strengths use. For love, curiosity, zest, hope and perseverance, there were not only a significant indirect but also a significant direct effect. Similarly, hope also had the highest direct effect on SWB with the regression weight 0.78^∗∗^. For the remaining character strengths, there were only a significant indirect effect and no significant direct effect, which means that the relationships between these character strengths factors and SWB were mediated by strengths use.

### The Moderated, Mediating Analysis

In order to assess the moderating role of future self-continuity in the link between character strengths and strengths use and the link between strengths use and SWB, we used the PROCESS macro written by Hayes (2013) to conduct moderated mediation analyses. This macro allowed us to get the estimates of the model and the conditional indirect effect and hypothesis tests conditioned on the moderators being set to the sample mean and ±1 SD and also can produce the conditional indirect effect at the different value of the moderator for which the effect is just statistically significant (at α = 0.05) using the J-N technique. Similarly, we z-transformed all the variables in order to compare effect sizes and reduce multicolinearity before this analysis.

As shown in **Table 5**, future self-continuity yielded no significant effects on strengths use and there was no significant interaction between character strengths on the whole and future self-continuity in the mediator variable model; whereas future self-continuity had the significant effect on SWB and the statistically significant interaction between strengths use and future self-continuity in the dependent variable model for SWB suggests that the indirect effect of character strengths on SWB through strengths use is moderated by future self-continuity. The positive sign of the interaction indicated that the indirect effect is large for students with higher level of future self-continuity. With the significant interaction, it allowed us to probe the indirect effect at different levels of the moderator. The default output displays the conditional indirect effect at three values of the moderator variable (the mean and ± 1 SD from the mean). As **Table 6** shown, three conditional indirect effects of future self-continuity were positive and significant. Specifically, when the value of the moderator was at one SD below the mean, the mean and above the mean, the indirect effect were significantly different from 0 at α = 0.05, yielded 95% BCa CIs of {0.0137, 0.0590}, {0.0322, 0.0709} and {0.00438, 0.0911}, respectively. In addition, the mediating effect of strengths use in the Moderated Mediation Model was also found to be significant (95% CI: {0.0019, 0.0193} with 5,000 resamples).

**Table 5:** Results of moderated mediation analyses for future self-continuity moderating strengths use’s mediation of character strengths and subjective well-being.

Predictor	*B*	*SE*	*t*	*p*
**Mediator variable model**			
CCS	0.064	0.005	12.4677	0.0000
CW	0.004	0.033	0.1213	0.9035
CCS × CW	–0.005	0.003	–1.6633	0.0977
**Dependent variable model**			
CCS	0.013	0.013	1.038	0.3006
CM	0.758	0.128	5.919	0.0000
CW	0.285	0.062	4.587	0.0000
CM × CW	0.165	0.063	2.615	0.0095

*CCS, Character Strengths; CM, Strengths use; CW, Future Self-continuity.*

**Table 6:** Results of conditional indirect effects of future self-continuity.

FSC score	Conditional effects at future self-continuity + 1 SD			
	a_1_(b_1_+ b_3_W)	*SE*	(Boot) LLCI	(Boot) ULCI
—1.00	0.0337	0.0115	0.0137	0.0590
0	0.0493	0.0097	0.0322	0.0709
+1.00	0.0648	0.0119	0.0438	0.0911

*The conditional indirect effect is computed a_*1*_ (b_*1*_ + b_*3*_ W) where a_*1*_ is the path from character strengths to strengths use, b_*1*_ is the path from strengths use to subjective well-being, b_*3*_ is the path from the interaction of strengths use and future self-continuity to subjective well-being, and W is future self-continuity.*

**FIGURE 1:**
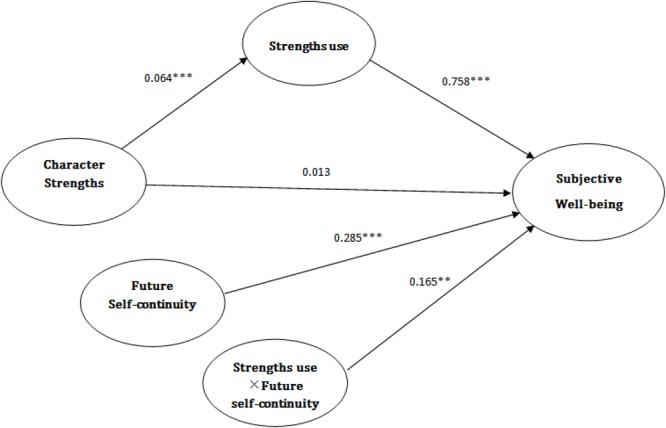
Moderated mediation model. The moderating effect of future self-continuity on the mediation of character strengths and subjective-well being by strengths use. ^∗^*p* < 0.05; ^∗∗^*p* < 0.01; ^∗∗∗^*p* < 0.001.

Overall, future self-continuity moderates the link between strength use and SWB, and this link was significant in three conditional indirect effects of future self-continuity (see **Figure 1**).

## General Discussion

This current research had two objectives: (1) to examine the mediating role of strengths use in the relationship between the 24 character strengths and SWB, and assess the indirect effects of each character strength on SWB through strengths use; (2) to explore the moderating role of future self-continuity in the link of character strengths and SWB and the link of strengths use and SWB. Additionally, the common-endorsement of character strengths and the associations between the 24 character strengths and SWB in the sample of Chinese undergraduate students were also investigated.

Firstly, four of the top five “SS” in the present sample were: fairness, love, authenticity, gratitude, and the least strengths included creativity, perspective, learning and regulation. This result was largely consistent with several studies conducted with samples of Chinese college students (Zhang, 2009; Li, 2015). For example, Zhang (2009) suggested that, for undergraduate students, authenticity, kindness, love and fairness were the most common character strengths whilst creativity, perspective, bravery, social and modest were the least ones. Moreover, Li (2015) found that the top 5 “SS” were: love, fairness, gratitude, teamwork and integrity whilst the bottom 5 were self-regulation, love of learning, perspective, creativity and social intelligence. Of these studies, it could be concluded that love, fairness, gratitude are relatively common SS while the strengths of creativity, perspective, love of learning and self-regulation ranked at relatively low positions among Chinese university students.

Furthermore, when compared to research conducted in other nations, results suggested there is a certain degree of similarity and consistency in terms of the most commonly endorsed and least character strengths. For instance, Park et al. (2006) found that the most commonly endorsed strengths from country to country included fairness and gratitude and the least strengths included self-regulation, and also the rank ordering of strengths across different nations showed certain degrees of similarity. To the extent, these analyses reveal that there is something similar in the endorsement of strengths across different nations and cultures, which could be seen as indicators about universal human nature. As Bok (1995) suggested, the universal values pervasively across nations and societies include reciprocity and positive obligations to care mutually and negative injunctions against cheat and betrayal as well as consistent standards of even-handedness and procedural justice in conflicting situations concerning positive obligations and/or negative injunctions. In the present study, the mostly common strengths of love and gratitude precisely reflect positive obligations, and the strength of authenticity embodies negative injunctions and the strength of fairness represents standards of fairness and procedural justice.

Nevertheless, that the most commonly endorsed strengths from nation to nation are universal does not indicate that people from different contexts have totally similar endorsements of strengths. As Shimai et al. (2006) suggested Americans were more likely to rank highly humor, perspective and integrity while Japanese ranked these strengths at relatively bottom position. These inconsistent results indicated that although there is something universal about the endorsement of personal strengths, the profile of character strengths certainly differs from culture to culture and from country to country. For example, the strength of bravery, in the western cultural system underlies “speaking up for what is right even if there is opposition,” and among the VIA, one of the items assessing this strength is “when I hear people say something mean, I make a protest.” That is, if people exhibit such behavior, then they may be seen as a person with the strength of bravery. However, it ought to be noted such behavior may not be appropriate in the Chinese context as Chinese people emphasize more importance of harmony, “mianzi” (namely, face) and tend to express their thoughts and emotions in a reserved way.

Additionally, analyses showed gender differences in the distribution of character strengths, that is, females were more likely to endorse higher scores on teamwork, fairness, Love and gratitude than males, whereas males were more likely to endorse higher scores on judgment. These differences were in line with the previous studies (Shimai et al., 2006; Linley et al., 2007; Brdar et al., 2011; Li, 2015). As Brdar et al. (2011) suggested, gender difference in character strengths may be explained from three different perspectives: (a) evolutionary perspective, that is, females are more likely to endorse strengths such as kindness and love possibly because such strengths are related to their natural, evolutionary roles as chief caregivers, while males are more likely to endorse strengths such as bravery possibly because of their natural, evolutionary role in hunting and looking for food to survive; (b) social construction perspective, that is, as males and females have distinct social roles, they develop different characteristics and traits. Specifically, males tend to develop characteristics such as self-reliance and invulnerability and to be tough and strong as they are more likely to be the backbone in the family, whereas females are more likely to be sympathetic and careful and sensitive that makes them greatly recognize others’ needs and response to others’ emotional expression; (c) biosocial perspective, that is, biosocial interactions including the evolutionary sex roles and developed social roles and some certain settings in society cause gender difference in terms of character and traits. To the extent, findings of the present study support the proposed arguments though we didn’t provide such evidence that males are more likely to endorse bravery (valor) and persistence (industriousness and perseverance). Nevertheless, it should be noted that apart from these strengths scored significantly different between males and females such as kindness, love, gratitude, there were more similarities of the endorsement of character strengths between genders. Therefore, gender difference in character strengths ought not to be exaggerated (Linley et al., 2007).

Secondly, the correlation analyses showed that the strengths of hope, curiosity, zest, perseverance and love were closely related to SWB after controlling for influences of students’ gender. This finding was largely consistent with prior studies (Park et al., 2004; Shimai et al., 2006; Brdar and Kashdan, 2010; Littman-Ovadia and Lavy, 2012; Proyer et al., 2014; Hausler et al., 2017). As Park et al. (2004) suggested, though on the whole having character strengths belonging to the classification of VIA are generally associated with psychological and subjective fulfilling, some strengths appear to be more important than others. In their survey, among the 24 character strengths the most robust associations with life satisfaction were love, zest, hope, and gratitude. Unsurprisingly, these positive strengths are indeed grounded in human nature, and contribute genuinely to “the good life” for individual.

According to Peterson and Seligman (2004), the strength of love, emphasizing good interpersonal relationship and mutual care and reciprocity, is the brilliant one that enables humans to achieve fulfillment and happiness; the strength of zest, manifesting strong vitality and enthusiasm for life, endows people with energy and excitement to approach colorful life; the strength of hope, a belief that a good future can be brought about, connects one to the future in a happy way; and lastly the strength of gratitude, being grateful to good things happening and expressing thanks, connects one to the past in a happy way. As well, Park and Peterson (2006a) also suggested that strengths of the heart such as gratitude, hope, love and zest were more related to well-being than are strengths of the head such as creativity and judgment. Furthermore, in the longitude study, Gillham et al. (2011) demonstrated the roles of transcendence strengths including hope, zest, love, gratitude (as well as meaning) in general happiness, life satisfaction and future well-being. Taken together, empirical research has supplied sufficient evidence for the unique and ubiquity benefits of these four distinct strengths and, arguably certain character strengths tend to be more important than others. These findings provide basic knowledge for developing intervention strategies aiming to improve individual well-being and happiness more efficiently and effectively.

Thirdly, the mediation analyses suggested that strengths use wholly mediates the relationship between character strengths on the whole and SWB. That means, the possession of strengths, as an essential condition, enables individual to display strengths-related behavior, and in turn, makes ones experience satisfaction and joy. In other words strengths use played a “bridge” between the possession of character strengths and SWB. Perhaps this finding would be seen as a psychological tautology as we think that we may have been developing the pattern that naturally endows us with ability to apply our own strengths that we possess, and experience satisfaction. However, such a process or pattern may not be so spontaneous and simple as the above mentioned. In many cases, people fail to achieve the active and comprehensive good life what they ought to not because they do not possess the strengths to a certain extent that can be greatly beneficial to achieve happiness but they do not show strength-related behavior. Similarly, James (1907) suggested that people the world over have amounts of resource, but most individuals make use of only a small part in the whole lifetime, while only a small group push to their extreme of use and thus exceptional. Therefore, a possible explanation of this present finding may be that possessing the more of the strengths (e.g., creativity) is a prerequisite of a fulfilling life, but the unblocked deployment of such strengths is a more direct predictor of achieving optimal functioning.

Consistent with this explanation, Murillo (2012) concludes that possessing good character and exercising relevant behavior in the right way are two essential conditions for the good life but ultimately happiness is the end to which our actions are directed. Empirical literature has also supported the important role of applying personal strengths (Botha and Mostert, 2014; Dubreuil et al., 2014; Littman-Ovadia et al., 2017), and the direct effect of strengths use on well-being (Govindji and Linley, 2007; Wood et al., 2011; Douglass and Duffy, 2015; Huber et al., 2017). Consistent with our finding, in the work of Govindji and Linley (2007), they also suggested that although strengths knowledge and strengths use were both highly correlated with indicators of well-being but the former would not cause any significant increases in well-being, whereas using strengths promoted greater well-being. Overall, our finding answered the proposed question “how strengths of character operate to well-being,” and uncovered the underlying process from character strengths to well-being. Indeed, it was through strengths use.

More particularly, the mediation analyses further examined the indirect effect of the 24 character strength on SWB through the “bridge” of strengths use. Results showed that strength use mediates the relationships between every strength and SWB and the indirect effects of strengths use varied from different strengths. Among these strengths, perspective and social yielded the highest indirect effects. Regarding this finding, possible reasons were that possessing more perspective helps people form a broader perspective and in turn, enables them to tackle their problems appropriately and also provide sensible advices for others, which may be beneficial to develop good interpersonal relationships, and in turn lead to greater PA and well-being; and possessing social intelligence enables them to understand the social world more easily and adapt to others’ feelings and expectations, which helps them behave and speak in right ways, and foster positive relationship with others, and thus, they are happier (Park et al., 2004). Thus, it could be inferred that developing and using strengths that relate to the good interpersonal relationship would be a pretty effective strategy to improve individual well-being and satisfaction. To some extent, this explanation is also consistent with the finding of the longest study on happiness conducted by the Harvard Study of Adult Development that good relationship is the secret of the good life.

Finally, regarding the second objective exploring the moderating role of future self-continuity in the link of character strengths and SWB and the link of strengths use and SWB, analyses demonstrated that future self-continuity moderates the relationship between strengths use and SWB, and this relationship was significant in all three conditional indirect effects of the moderator. Indeed, this is an original finding of such a role of future self-continuity in the link between strengths use and SWB, but previous literature demonstrated the perception of self-continuity as an important predictor of psychological adjustment (i.e., SWB) and equanimity (Landau et al., 2008; Sani et al., 2008; Timothy et al., 2011).

Further, in the discussion of self-continuity already research suggested that, with regard to the perceived connectedness and persistence from past to present, when individuals recall positive events such as making the right choice, engaging in effective behavior and perceive these event in accordance with their self-concept, positive emotional responses would be provoked, and the stronger the self-concept, the greater intensity of positive affection provoked. From this perspective, people may have certain emotional responses when anticipating their future selves (like recalling of their personal past) (Ritchie et al., 2016). One might feel ambitious when anticipating great success they may achieve; one might feel pleased when thinking about the benefits they will get; and one might be satisfied when considering good consequences in the near or relatively distant future caused by their current behavior. Thus, a possible explanation of the present finding is that using their strengths as engaging behavior makes people feel as if he can reveal his true self, being more authentic and vigorous and in turns resulting in optimal functioning, and for those endorsing a sense of continuity and connectedness with a future self, they are more likely to experience joy through anticipating beneficial outcomes of their present action. Nevertheless, it is also possible that the links of these variables on well-being are influenced by some other underlying processes such as goals pursuit and mental imagery and that the moderating effect of future self-continuity is a mere epiphenomenon. Therefore, it is necessary for future studies to investigate more underlying mechanisms of these associations by considering other variables related to future self-continuity and well-being.

### Limitations and Future Directions

The present research has several limitations. First, this was a cross-sectional study, and thus the obtained results could not demonstrate casual relationships among these variables involved. Moreover, research on mediation analyses showed that complete mediation effects examined by cross-sectional data might be non-significant in a longitudinal study (Maxwell and Cole, 2007). That means the mediating effect of strengths use demonstrated in the present study might be biased. However, according to the longitudinal research conducted by Wood et al. (2011), strengths use indeed predicted the improvement in well-being and optimal functioning over time, and the positive relation between character strengths and well-being has been well-established by existing longitudinal studies (Shoshani and Slone, 2013; Duan and Ho, 2017). While these studies have provided empirical evidence for the model the present study constructed, future research is needed to further examine the mediation effect with longitudinal data. Furthermore, the present study adopted participants’ composite scores of strengths use, instead of allowing them to identify their each strength and then asked about the deployment of specific strength. It is possible that the latter measurement approach is a more effective way to find stronger relations between character strengths, strengths use and well-being outcomes.

Second, the small sample size raised questions about how representative participants are of others engaged in similar educational context. Future research is needed to investigate the relations between character strengths, strengths use, future self-continuity and SWB to the wider population, and to establish the ubiquity of the present findings. Third, the present study adopted self-reported instruments to measure all variables involved, and thus the common method variance (CMV) would be a considerable concern. Although we have used the pre-control method including adopting the same instructions informing participants that there was no right or wrong answer and ensuring the anonymity of participants, which may reduce CMV to the extent, we still encourage future studies to adopt more appropriate designs and multiple approaches to reduce such bias such as changing scales types and arranging items in varying order (Zeng et al., 2016).

Fourth, the present study considered SWB as the outcome variable, and adopted Diener’s (1984) model of SWB containing positive affect, negative affect, and life satisfaction to measure SWB because of its wide acceptance and well-established generalizability. However, it is suggested that for better understanding of multi-facet constructs of well-being and its relations to character strengths and strengths use as well as future self-continuity, future research should adopt some other models of well-being such as Ryff’s (1989) model of PWB and Seligman’s (2011) PERMA of well-being. Finally, given that this was the original study to present the moderating role of future self-continuity, future research should further investigate whether there are more underlying mechanisms (or other psychological or behavior variables such as goals pursuit and mental imagery) in such relations, which would provide more specific evidence to elucidate the role of future self-continuity in relation to strengths use and well-being.

### Implication

The findings of the current research have several practical implications. Firstly, the obtained results demonstrated strengths use plays a bridge between possessing strengths and SWB. To the extent, that means, possessing the more of the strengths is a prerequisite of a fulfilling life, and the unblocked deployment of such strengths is the direct predictor of achieving optimal functioning. Therefore, educators, teachers and school coaches should encourage students not only to identify their strengths but also apply their strengths. Also, the schooling should present more opportunities for youth to display strength-related behavior. It would be beneficial for students to engage these effective behaviors, resulting in optimal functioning and experiencing great happiness. Secondly, considering the role of future self-continuity, existing literature, coupled with the current research, suggested that a feeling of connectedness to the future self could be vital for people in various domains of life such as time management, decision making, academic performance, coping and well-being. More importantly, in the education context, it is necessary for students to be endowed with a strong sense of future self-continuity that allows them to realize the continuing nature of self and makes them show more appropriate and sensible behavior and prepare them to anticipate the positive consequences of such action in the long term and in turn feel engaged and energized. Overall, these findings demonstrate sights for the current and future educational programs, considering strengths use and future self-continuity promising factors of personal and positive functioning and further suggest recommendations for well-being interventions in the educational setting.

## Conclusion

The present study extended the existing knowledge of the relation between character strengths and SWB by adding to strength use as a mediator. Findings suggested that strengths use plays a bridge between character strengths and SWB, and the indirect effect mediated by strengths use varied from different character strengths. Moreover, the study also examined the moderating effect of future self-continuity in the relation of character strengths, strengths use and SWB, and results showed that future self-continuity moderated the link of strengths use and SWB. This finding implied that the strategy of focusing on the future self may play a key role in educational context. That is, the continuous perspective of the present -to-future selves may enable students to think more about positive consequences of their current actions such as devoting more effort to their studying in the long term and in turn experience greater joy. This would not only cause positive consequences of “traditional skills” but also lead to “happiness,” in accordance with the core of positive education.

## Ethics Statement

The study was carried out in accordance with the recommendations of ‘the University of Southwest’s Human Research Ethics Committee’ with written informed consent from all subjects. All subjects gave written informed consent in accordance with the Declaration of Helsinki. The protocol was approved by the ‘the University of Southwest’s Human Research Ethics Committee.’

## Author Contributions

YZ and MC designed the experiments, carried out the experiments, and wrote the manuscript. MC analyzed the experimental results.

## Conflict of Interest Statement

The authors declare that the research was conducted in the absence of any commercial or financial relationships that could be construed as a potential conflict of interest.
